# The global biopharma industry and the rise of Indian drug multinationals: implications for Australian generics policy

**DOI:** 10.1186/1743-8462-4-10

**Published:** 2007-06-01

**Authors:** Hans Löfgren

**Affiliations:** 1 School of International and Political Studies, Deakin University, Melbourne, Australia.

## Abstract

This article provides a synopsis of the new dynamics of the global biopharma industry. The emergence of global generics companies with capabilities approximating those of 'big pharma' has accelerated the blurring of boundaries between the innovator and generics sectors. Biotechnology-based products form a large and growing segment of prescription drug markets and regulatory pathways for biogenerics are imminent. Indian biopharma multinationals with large-scale efficient manufacturing plants and growing R&D capabilities are now major suppliers of Active Pharmaceutical Ingredients (APIs) and generic drugs across both developed and developing countries. In response to generic competition, innovator companies employ a range of life cycle management techniques, including the launch of 'authorised generics'. The generics segment in Australia will see high growth rates in coming years but the prospect for local manufacturing is bleak. The availability of cheap generics in international markets has put pressure on Pharmaceutical Benefits Scheme (PBS) pricing arrangements, and a new policy direction was announced in November 2006. Lower generics prices will have a negative impact on some incumbent suppliers but industrial renewal policies for the medicines industry in Australia are better focused on higher value R&D activities and niche manufacturing of sophisticated products.

## Background

The term biopharma captures a broadening of discussion about pharmaceutical policy and the growing significance of biotechnology. Indeed, the commercialisation of the discoveries of the biological sciences, oriented towards an understanding of living cells at the molecular level, is widely seen as potentially sustaining another long wave of economic growth, 'making reality of the prediction that this will be the century of biotechnology' [[1], p. 5, see also [2]]. The long-established 'big pharma' companies headquartered in the US and Europe – roughly those listed in *Pharmaceutical Executive's *annual company ranking – are in the midst of 'an extremely painful transition' driven by a confluence of scientific-technological, economic and regulatory-political factors [3,10]. Generic competition has led many of these firms into take-overs and mergers, and a flurry of alliances and other agreements with major generic players. Pricing pressures, safety recalls and marketing scandals also challenge an industry which has, for decades, been enormously profitable [4,5].

The industry's research and development (R&D) productivity crisis is a crucial factor driving new business strategies. In recent years, established R&D systems have seen only a trickle of new drugs emerging. The 20 new molecular entities approved by the Food and Drug Administration (FDA) in 2005 were fewer than half of the number approved in 1996 [6]. This would not be such a problem if many of the new products were of major therapeutic and commercial significance. But most newly launched drugs provide marginal health benefits only, and commercial returns are then constrained by the cost-effectiveness hurdles imposed by public and private purchasing organisations. A French study reports that '68 percent of the 3,096 new products approved in France between 1981 and 2004 brought "nothing new" to existing treatments' [7].

Some new drugs, of course, do bring significant therapeutic benefits, and biotechnology has opened up promising new avenues for drug discovery. Sales of biologicals (biotechnology-based drugs) in 2005 grew about three times faster than the small molecule market, reaching about US$52 billion. Biologicals are reported to account for 27% of new medicines under development, but the prospect of a wave of new break-through medicines, comparable to the synthetic drugs of post-1945 decades, has not yet been realised [8-11]. 'Big pharma' companies have responded to cost and profit pressures with consolidation into ever larger entities, combined with outsourcing, and other types of exchange, with external organisations [12-14]. The result is a global pattern of production and innovation networks encompassing linkages between 'big pharma' and smaller R&D intensive firms and a variety of new commercial players, such as clinical research organisations, some of which (for example Quintiles) are close to billion dollar companies in their own right [15]. Agreements with generics firms, to thwart the full impact of competition in off-patent markets, are also on the rise.

The US remains the central node of these networks. Public funding for medical research is of greater magnitude in the US than anywhere else; the world's largest medical research funding organisation, the National Institutes of Health, has a budget of around US$28 billion. Half of the top 10 pharma companies are headquartered in Europe – GlaxoSmithKline, Sanofi-Aventis, Novartis, AstraZeneca and Roche – but a significant proportion of their R&D is undertaken in the US. The significance of India, China, South Korea and other Asian countries is growing [10]. As detailed further below, Indian firms have achieved a position as significant players in global biopharma markets. In particular, low costs coupled with high quality personnel make Indian firms competitive suppliers of manufacturing and R&D services, and other outsourced activities. In some areas, notably in the production of Active Pharmaceutical Ingredients (APIs), there are now no technology gaps between Indian and Western firms [16,17]. The credibility of Indian drug firms has benefited from the reputation of Indian companies in the global market for information technology-based Business Process Outsourcing (BPO) services [18,19]. China is considered to be ahead of India in biology and to have made notable advances in gene therapy and stem cell research, but the top tier of the Indian drug companies such as Ranbaxy and Dr Reddy's Laboratories (DRL), which also have extensive biotechnology capabilities, have a stronger presence in global markets.

Present changes to Australian drug pricing arrangements (see the editorial accompanying this article) are driven by concerns that the Pharmaceutical Benefits Scheme (PBS) provides for unnecessarily high prices for generic products. This contention is confirmed in the following analysis, which explores the complexities of the global generics sector and elucidates the context for Australian medicines policy.

## Generics and global industry dynamics

The modern generic pharmaceutical industry came into existence through the 1984 US Drug Price Competition and Patent Restoration (Hatch-Waxman) Act, which provided for facilitated market entry for generic versions of all post-1962 approved products in exchange for an extension of the patent period for the original drug [20,21]. As more generics became available, US health maintenance organisations and pharmacy benefit management companies encouraged or mandated measures such as generic prescribing, brand substitution by pharmacists, and reimbursement on the basis of cheapest brand. In 2005, more than 60% of prescriptions in the US were filled with a generic. Their established role in the US effectively debunks the disparagement of generics that is still occasionally forthcoming from brand industry sources such as the Pharmaceutical Research and Manufacturers of America (PhRMA) [22]. Other countries with highly developed generics markets include the UK, Germany, the Netherlands, Canada, and the Nordic countries, and generics markets are expanding rapidly, from a lower base, in France, Spain, Italy, Russia, Latin America, Australia, and elsewhere [23].

As patents expire on many big-selling products, 'about $100 billion worth of brand-name drugs will lose patent exclusivity [in the US] during the next five years, including products going off patent in 2006 that will generate about $21 billion in combined sales' [24]. Recent examples include Pfizer's antidepressant Zoloft (sertraline) and Merck's anti cholesterol drug Zocor (simvastatin), which both lost US patent protection in June 2006. The world's largest generics company, Teva, headquartered in Israel, and the Indian firm Ranbaxy (tenth in global generics rankings) obtained 180 days US exclusivity for generic simvastatin of various strengths. The availability of generic simvastatin has flow-on implications across the anti-cholesterol market, which in 2004 had a global value of about US$25 billion [25]. In Australia, generic brands of simvastatin were first listed on the PBS on 1 August 2005. It was envisaged initially that the reference pricing system would trigger a price reduction for all brands in the statin group of drugs, but the PBAC accepted a submission from Pfizer for Lipitor to be excluded from the price cut on the grounds of being ' more effective than simvastatin in lowering cholesterol' [26].

Plavix, one of the world's highest selling drugs at about US$4 billion in 2005, marketed in the US by Bristol-Myers Squibb (BMS) for Sanofi-Aventis, is another recent example of a major product going off patent. In August 2006, the Canadian company Apotex after complex legal wranglings launched a generic version of Plavix. Within days, close to 80% of new prescriptions in the US were filled with the Apotex generic, and BMS and Sanofi-Aventis lost around US10 billion in market value [27,28].

The next big generics development is the introduction of regulatory pathways for biogenerics. These are generic versions of biotechnology-based drugs (biopharmaceuticals or biologics) such as insulin and human growth hormone, that is, large-protein molecules derived from living cells. Biogenerics involve a substantial innovative input, and are therefore not strictly generic medicines in the traditional regulatory sense, and are sometimes referred to as biosimilars or follow-on protein products. But they are sufficiently similar to a product already approved to make substitution possible. The cost of the original biologics is typically well in excess of US$10,000 per patient per year, and this market is growing much faster than sales of traditional drugs. In 2005 the value of biopharmaceuticals in the US was around US$30 billion [29]. Australia's Therapeutic Goods Administration (TGA) was the first regulatory agency, in 2004, to approve a biogeneric, namely Omnitrope for treatment of growth disorders, supplied by Sandoz. Omnitrope has since been approved also in the European Union and the US.

The complexity of biogenerics manufacturing, and regulatory uncertainties, have so far protected originator products from competition even when patents have expired. Regulatory processes for biogenerics are not yet streamlined but the case of Omnitrope is a sign of things to come [30]. Several companies including Indian firms stand ready to launch biogenerics. Technical barriers to entry make this market, if anything, more appealing to generics players with requisite technological capabilities. Major originator products are expected to give rise to only one or two biogenerics, likely to be priced 25 to 40% below the original. This translates into profit margins much higher than in generic small-molecule markets where price falls tend to be much steeper [31,32].

To counteract the threat of generic competition, 'big pharma' applies a range of sophisticated 'life cycle management' techniques, notably the 'evergreening' of patent protection through the embedding of brands in intricate clusters of patents which make challenges complex and expensive [15,33]. Other techniques include the packaging of two existing drugs, about to go off patent, into a single new patented drug. For example, Pfizer's Caduet, containing both Lipitor and Norvasc, is expected to retain patent protection in the US until 2018, long after competition has eroded the value of its basic chemical ingredients [[5], p. 72]. The patenting of a marginally modified version of the original chemical substance, coupled with massive marketing campaigns to switch patients to the newly patented product, has also proven commercially effective. The industry standard in this type of strategy was set by AstraZeneca which successfully launched Nexium when Losec/Prolosec was about to lose patent protection. Nexium 'has been found to be pretty much identical to Prilosec and about ten times more expensive' [[5], p. 76]. Other ways of managing the 'life cycle' of drugs include new delivery technologies (such as once-a-day formulations), new indications which may bring additional periods of exclusivity, switch to over-the-counter (OCT) status, and so-called 'exclusion payments' made 'when a branded company shares a portion of its future profits with a potential generic entrant in exchange for the generic's agreement not to market its product' [[23,34], p. 6].

## Blurring of originator and generic categories

This complex environment leaves little scope for 'traditional' generics companies competing solely on price. Indeed, mergers and acquisitions have brought forth a small number of multinational generics majors, with technological, legal, and marketing capabilities approximating those of 'big pharma'. Firms in this group include Teva, Mylan Laboratories, Actavis, Barr (which in 2006 acquired Pliva, the largest drug company in Eastern and Central Europe), Watson (recently merged with Andrx, another generics major), and the Indian companies Ranbaxy and DRL. These are companies dealing also in proprietary products such a new drug delivery technologies, and in some cases they engage in the development of new innovative drugs [23]. Merck KGaA, the parent company of Alphapharm in Australia, is a long-standing member of this group, but has declared a full-scale shift into the innovator category (following the acquisition of Serono, a large biotech company) and its generics division has been off-loaded the US-based generics major Mylan Laboratories. The blurring between the brand-name (originator, innovative) and generics sectors is demonstrated most starkly by Sandoz, which ranks globally as the second largest of the generics suppliers (after Teva). Sandoz was established by Novartis (the world's fourth largest pharma company) in 2002 from a collection of previously existing subsidiaries. Similarly, Sanofi-Aventis has created Winthrop Pharmaceuticals 'to position the Group ... as a major actor on the generics market' [35]. It makes good sense for Novartis and Sanofi-Aventis to supplement the core business of patented products with a strong presence also in the generics market. In the US and elsewhere, a capacity to supply an extensive range of products, including off-patent drugs, can be a competitive advantage.

The phenomenon of authorised generics, also known as pseudo-generics or fighting-brands, represents an intriguing form of life cycle management. This refers to brand-name products given a generic label; the 'brand-name manufacturer either sell [s] the authorized generic itself through a subsidiary or licens [es] a generic firm to sell the authorized generic' [[34], p. 27]. This practice is the subject of heated debate in the US and is viewed by the Federal Trade Commission as potentially illegal anti-competitive conduct. Pseudo-generics can be harmful to consumers 'because an expectation of competition from authorized generics will significantly decrease the incentives of generic manufacturers to pursue entry prior to patent expiration [through patent challenges], especially for "non-blockbuster" drugs' [[34], p. 28]. Authorised generics would seem to have become common also in Australia but a detailed analysis of their impact on local market dynamics is yet to be undertaken [but see [36]].

Patent expiries and the growth of the generics sector is only one of the factors which explain the changing dynamics of the global biopharma industry. A major trend is the expansion of outsourcing by the major companies across a range of functional areas, including manufacturing, and R&D and clinical trials. It is striking that Asian and in particular Indian firms are now highly competitive providers of outsourcing services including supply of APIs [3,17,22,23,37,38].

## India's biopharma multinationals

India's large biopharma industry is one of the country's economic success stories. Some Indian drug companies were established well before the Second World War. The modern drug industry emerged however from the 1970s, behind high tariffs and through other protectionist measures [39,40]. In 1972, patents for pharmaceutical (and food and agrochemical) products were disallowed and only one production process could be patented (for a maximum seven years). Indian firms proved capable, often through collaborations with public sector research organisations, of developing alternative processes for the production of most APIs and generics, drawing on a strong chemical engineering tradition.

The industry has now reached a scale and scientific-technological sophistication which make Indian firms important global players. In volume terms, India's drug industry is the fourth largest in the world; around 20% of the global production of APIs and formulations is estimated to derive from Indian manufacturing plants [41]. The domestic market is, along with Japan's, unique in not being dominated by 'big pharma'. In 2005, Indian firms were reported to meet 'around 70% of the domestic demand for bulk drugs, drug intermediates, pharmaceutical formulations, chemicals, tablets, capsules, orals and injectables' [[42], p. 10]. The competitiveness of top-tier firms draws on an abundant supply of engineering and science graduates and 'the world's second largest annual pool of English-speaking medical professionals after the US' [[43], p. 57]. For lower pay than an equivalent professional in the US, 'a full-time chemist in India is better educated ... [and] the time spent on the job is longer' [[44], p. 198]. Indian drug have been exported to developing countries for several decades, and more recently exports to North America and Europe have increased rapidly. With marketing infrastructure, production plants and R&D facilities in Europe and North America, and a long-standing presence in many developing countries, the top companies are true multinationals. A recent wave of overseas acquisitions, notably several medium-sized local generics firms across Europe, consolidates their presence in international markets [45]. Ranbaxy, which launched its first PBS products in 2006, claims to be 'no longer an Indian company': ' [o]ur origins are in India, but we are truly a global pharmaceutical company' [46].

The Trade Related Intellectual Property Rights (TRIPS) agreement of 1995 has been a key driver of the outward orientation of Indian drug firms. TRIPS, one of the three pillars of the World Trade Organization (WTO), the others being trade in goods and services, mandated the introduction of globally harmonised intellectual property rights (IPRs). India was given a ten year transition period, and in 2005 took the final step in making its IPR regime TRIPS compliant. The most essential aspect of the new patent system is the disallowance of the 'reverse engineering' model which underpinned the drug industry's expansion in the preceding three decades. The industry initially had deep misgivings about TRIPS, which removed its major competitive advantage, but the leading companies have now adapted their business strategies to the new regulatory environment [39,40,47,48]. Apprehensions today are expressed not so much by the industry but by health activists and non-government organisations, and focus on the implications of TRIPS for affordable drug access for the poor in India, and in other developing countries which have hitherto been able to import relatively cheap generics from India. Most already marketed generic medicines will continue to be available under TRIPS, but it prevents the production and exports of generic versions of new patented drugs, for example any new break-through HIV/AIDS medications, unless compulsory licensed, or voluntarily licensed by the patent holder [49,50].

Until recently, Indian firms expended only 2–3% of revenue on R&D, and drug discovery research was undertaken in public sector research institutes only, but the last decade has seen a steady rise in private sector R&D, spurred by the new IPR regime. In 2006 more than 175 companies had established R&D centres recognised by the Department of Scientific & Industrial Research. Around 15 companies are reported to be engaged in discovery research, spending around 10% of revenue on R&D [51]. Ranbaxy, DRL, Nicholas Piramal, and several other firms, have molecules in advanced stages of clinical testing, and the industry in total is reported to have about 60 compounds in various phases of development though no company has yet brought a drug to market [52]. The industry's total annual R&D investment is estimated at around US$170 million, which is miniscule compared to that of 'big pharma', though this expenditure 'buys' more R&D in India than in North America or Europe due to lower labour costs and perhaps harder efforts [53].

Rather than head-on competition, alliances with 'big pharma' and the global generics companies form the primary growth strategy for the leading Indian companies. The resources required to take a promising molecule through testing and regulatory approval, and then the costs of marketing in Western countries, are of such magnitude that out-licensing to 'big pharma' is the predominant model [39]. As noted, the established global companies now outsource to India a range of activities including manufacturing and packaging, discovery research, clinical trials, and data management. Global biopharma outsourcing was valued in 2005 at around $60 billion, half of which encompassing R&D activities including clinical trials [54]. Clinical Research Organisations (CROs) in India are rapidly expanding and were reported in 2005 to be worth around $75 million [14]. India offers many advantages: a vast population of patients with a diverse gene pool, easily recruited and with little previous contact with modern medicine; availability of qualified English-speaking doctors, pharmacists and science graduates; a large number of hospitals and other institutions of good quality; and now protection for intellectual property. The cost of conducting clinical trials in India is estimated at 'almost one-third of the costs of the equivalent in the Western world' [44].

Large-scale, efficient manufacturing remains India's core competitive advantage in the biopharma sector. Around 75 plants are approved by the US Food and Drug Administration (the largest number of approved units outside the US) and many have Good Manufacturing Practice (GMP) clearance from Australia's Therapeutic Goods Administration (TGA) [14]. The marketing by Indian firms of their own products in Western markets is still at an early stage, though the chemical ingredients in many products carrying 'big pharma' brand names, or the labels of international or local Australian generics suppliers, are produced in Indian or other Asian plants. 'Big pharma' have always sourced APIs from European manufacturers but Chinese and Indian firms, drawing on a lower cost base, have emerged as major API suppliers. In 2005, India's API industry was the world's third largest, after China and Italy, growing at an annual rate of close to 20% [16]. Indian producers supply sophisticated APIs including still patented products to a greater extent than Chinse firms, which dominate in the market for off-patent commodity bulk drugs [16,17]. API costs, typically representing 40–50% of the value of generic oral solids, are critical to competitiveness in the generics industry [55,56].

India's biopharma innovation system lags behind the developed countries and even the largest of India's drug firms, Ranbaxy, is not ranked among the world's top 50 pharma companies [10]. But the entry of Indian firms into developed country generics markets has reinforced competition and price pressures; global 'pricing competition [is] driven by a huge overcapacity ... because of all the investments that have been made in India and are going to be made in China' [57]. Indicative of the confidence of Indian manufacturers is the claim by a Hyderabad-based company that it has the capacity to supply the US generics market, with a 30–40% margin, even after a 97% price collapse following patent expiry (personal communication, April 2006). Clearly, changes in international generics market, particularly the rise of Indian suppliers, provide a context for consideration of options for Australian PBS reform.

## Conclusion: implications for Australia

There are implications for two of the core aims of Australia's well respected national medicines policy flowing from the preceding sketch of developments in international markets. First, 'access to medicines', that is, the future of the PBS and, second, a 'responsible and viable industry in Australia', that is, the promotion of business activity in the biopharma sector [58].

Plainly, the expiry of many drug patents, and the strengthening of the global generics sector, opens up opportunities for PBS measures to extend the use of cheaper generics. This is a recognition which underpins the policy initiatives announced in November 2006, discussed in the adjoining editorial. It no longer makes sense to accept large gaps between PBS generics prices, and the prices at which many products can be sourced in international markets. The Department of Health and Ageing has long been aware of this discrepancy, highlighted in a March 2005 press release which compared prices for several off-patent products in Australia, the UK and New Zealand. In the case of fluoxetine 20 mg, the PBS price was reported to be $33.03, the price in the UK $4.97 and in New Zealand $1.45 [59]. It has also irked the government that a large proportion of costs benefits from cheaper generics have flowed to retail pharmacists rather than tax payers, an anomaly addressed in the policy package about to be presented to parliament [60,61]. In 2006 the introduction of a tendering system was reported to be under consideration but this idea was abandoned in the face of opposition from both suppliers and retail pharmacists [62].

Promotion of a local generics industry has figured as a policy aim within the context of the Pharmaceuticals Industry Action Agenda, pursued by the Department of Industry, Tourism and Resources since 2001. But this objective would seem to have been progressively downgraded for good reasons. The blurring of boundaries between the originator and generics sectors, internationally and in Australia, as outlined in this paper, make the notion of a distinct generics industry largely obsolete. The Australian generics market will undoubtedly see high growth rates in coming years: it is anticipated, even before any new legislation, that the present level of 25% of prescriptions filled with a generic 'will almost double in the next five years as more than half of the top 100 selling prescription medicines in Australia will be off patent and be substituted by generic branded drugs'. By 2009, it is expected that 'over 200 generic drugs will account for a dispensed value of approximately $1.7 billion' [[63], p. 12]. This does not translate however into good prospects for a generics manufacturing industry in Australia. The final stage of manufacturing and packaging of generics is essentially low-tech commodity production and, as in other areas of low-tech manufacturing, Australian-based plants are unlikely to be internationally competitive. The scale of generics manufacturing at individual production plants in India (and indeed China) is well beyond anything that can be envisaged for Australia. Recent entrants to the Australian generics market such as Genepharm source all products from overseas and this is the way of the future.

The policy measures announced in November 2006 will hurt incumbent generics suppliers, but broader negative economic effects will be marginal since Australia does not have significant manufacturing of either off-patent APIs or generic formulations. The structure of the Australian generics industry is highly skewed in favour of two companies; Alphapharm (now a subsidiary of Mylan Laboratories) and Sigma Pharmaceuticals control more than 80% of the market. The proliferation of authorised generics and shadowy discount practices weaken any case for protection of incumbent suppliers against competition from Indian and other new entrants. As noted, global industry dynamics provide little scope for Australia to become a significant drug manufacturing centre, with many generics heading towards commodity status. The scale advantages of manufacturers in India and elsewhere are such that Australian policy makers should instead focus on R&D-intensive biopharma sector activities, particularly in the biotech area where Australia retains a competitive advantage [64].

## Competing interests

The author declares that he has no competing interests.
